# A Study on the Establishment of Diagnostic Reference Levels for Cardiovascular Angiography and Interventional Procedures: Korean General Hospital

**DOI:** 10.3390/diagnostics16081243

**Published:** 2026-04-21

**Authors:** Daeho Kim, Jungsu Kim

**Affiliations:** 1 Medical Convergence Radiologic-Technology, Department of Bio-Health Convergence, Graduate School of Health Technology, Daegu Health College, Daegu 41453, Republic of Korea; hyaena4449@naver.com; 2 Department of Radiology, Daegu Fatima Hospital, Daegu 41199, Republic of Korea; 3 Department of Radiological-Technology, Daegu Health College, Daegu 41453, Republic of Korea

**Keywords:** diagnostic reference levels, cardiovascular intervention, cardiovascular angiography, kerma-area product

## Abstract

**Background/Objectives**: Cardiovascular interventions require prolonged fluoroscopy, which increases the risk of radiation. Diagnostic Reference Levels (DRLs), set at the 75th percentile of the dose distribution, are vital benchmarks for dose optimization. Following the release of national DRLs by the Korea Disease Control and Prevention Agency in March 2025, this study established institutional DRLs at a tertiary center to evaluate local optimization against national and international standards. **Methods**: This study analyzed radiation doses from 2022 to 2024 using DICOM Radiation Dose Structured Reports data from a single center’s angiography system. The total kerma-area product values and fluoroscopy times were evaluated across the categorized procedures. Following the International Commission on Radiological Protection guidelines, institutional DRLs were established at the 75th percentile of the dose distribution to benchmark against national and international DRLs. **Results**: Analysis of 1663 radiation dose structured reports established institutional DRLs, with the total kerma-area product ranging from 23.43 Gy·cm^2^ for coronary angiography to 329.45 Gy·cm^2^ for chronic total occlusion interventions. Complexity significantly increased the radiation burden; multivessel percutaneous coronary intervention and acute myocardial infarction nearly doubled the doses and fluoroscopy times in single-vessel interventions. Although the diagnostic procedures were cine image-driven, for moderate-complexity interventions, the contribution of fluoroscopy was greater. **Conclusions**: These findings support institutional optimization and development of safety guidelines to enhance patient protection during high-complexity cardiovascular procedures.

## 1. Introduction

In modern medicine, cardiovascular interventional procedures have become indispensable for the diagnosis and treatment of ischemic heart disease. In particular, coronary angiography (CAG) and percutaneous coronary intervention (PCI) have significantly contributed to improved survival rates and quality of life of patients with angina and myocardial infarction [1].

As the prevalence of cardiovascular diseases continues to rise because of an aging population and an increase in chronic diseases in South Korea, the number of related radiological examinations and interventional procedures has shown a corresponding, steady growth [2].

Medical radiation is indispensable for diagnosis and treatment. However, because of its inherent risks, its use requires both justification and optimization [3]. Patient exposure is permitted if the medical benefits outweigh the risks, and as such, legal dose limits do not apply to medical exposure. In this context, Diagnostic Reference Levels (DRLs) serve as critical tools for the prevention of unnecessary overexposure and optimization of radiation protection. DRLs are established on the basis of dose distribution data collected from standard-sized patients during commonly performed imaging examinations, and are typically calculated using the 75th percentile across medical institutions. Rather than acting as a mandatory regulatory limit, DRLs function as advisory benchmarks. They help medical institutions monitor patient doses and identify opportunities for improvement while maintaining diagnostic image quality. The goal of DRLs is not unconditional radiation dose minimization, but the achievement of an optimal dose level that ensures the acquisition of clinically necessary diagnostic information. This represents practical implementation of the fundamental principles of radiation protection, including keeping doses as low as reasonably achievable and dose optimization [4]. National and regional DRLs are based on patient data obtained through national surveys or registries and should be reviewed every 3–5 years. More frequent updates are required in the event of significant changes such as technological advancements, introduction of new imaging protocols, or improvements in post-processing techniques. If the dose indicators of an institution exceed the DRLs, a systematic review should be conducted, beginning with equipment performance evaluation followed by examination protocol and operator technique assessments. In particular, in fluoroscopy-guided interventional procedures, the air kerma-area product (KAP) and reference air kerma are recommended as primary DRL indicators, with the fluoroscopy time and number of acquired images serving as supplementary metrics. Whenever possible, DRL indicators should use values that are directly measurable during the procedure [5].

Cardiovascular interventional procedures have become the cornerstone of cardiovascular disease treatment, facilitating revascularization through the expansion of stenotic vessels or stent placement under angiographic guidance. However, the diagnosis and treatment of lesions within complex vascular structures often require prolonged fluoroscopic exposure, inherently exposing patients to significant levels of radiation [6].

Although X-ray guidance is indispensable for visualizing internal structures, the associated radiation exposure poses potential health risks to human tissue [7,8]. Of particular concern is the cumulative dose of medical radiation, which increases the probability of stochastic effects such as carcinogenesis [9]. This risk is exacerbated in cases involving complex multivessel disease, where an extended procedural duration or the need for repeated interventions can lead to substantially higher cumulative doses [10].

Consequently, maintaining the clinical efficacy of cardiovascular interventions while simultaneously minimizing and safely managing patient radiation exposure is a crucial ethical and medical responsibility for healthcare professionals.

To address these concerns, DRLs have been globally adopted as a fundamental instrument for radiation dose management [11,12]. International bodies, including the International Atomic Energy Agency and the European Union, advocate the establishment of national DRLs adapted to local clinical environments [13]. By enabling the identification of anomalously high doses, DRLs can encourage institutions to voluntarily benchmark their performance and implement systematic quality improvement strategies.

In March 2025, the Korea Disease Control and Prevention Agency released the first national DRLs specifically for cardiovascular interventional procedures in Korea, marking a milestone in domestic radiation safety management [14].

Significant variations exist in DRLs across different countries and continents. For instance, the reported DRL for CAG is approximately 35 Gy·cm^2^ in Europe, whereas it is as high as 83 Gy·cm^2^ in North America [15]. These discrepancies result from a complex interplay of factors, including national healthcare systems, the availability of advanced equipment, procedural protocols, and economic variables. Consequently, application of DRLs from other countries without modification is impractical. This necessitates the establishment of local or institutional DRLs that account for the specificities of the regional medical environment. As local DRLs directly reflect the performance of specific equipment, standardized protocols, staff proficiency, and primary patient demographics, they allow for more precise analysis and rapid corrective actions. In particular, use of a dose management system enables institutions to establish and update local DRLs at shorter intervals than those prescribed by national standards, thereby ensuring a flexible response to technological advancements and shifts in the clinical environment and, consequently, facilitating continuous radiation dose management and improvement. Ultimately, the effort to optimize radiation doses is an essential task that must be directly implemented by each medical institution at the point of care.

In this context, the aim of this study was to establish institutional DRLs for cardiovascular interventional procedures at a single tertiary center. By benchmarking these local values against the newly established 2025 Korean national DRLs and existing international standards, we validated the utility of institutional DRLs as an effective tool for continuous radiation dose optimization.

## 2. Materials and Methods

### 2.1. Study Design and Data Collection

This retrospective study was conducted to analyze radiation exposure doses in patients undergoing cardiovascular angiography and interventional procedures at a single medical center. On the basis of this analysis, we aimed to establish institutional DRLs and compare them with existing national and international DRLs. Data were collected for procedures performed between 1 January 2022, and 31 December 2024. The study population included patients who underwent cardiovascular procedures using the Azurion 7 M12 system (Philips Healthcare, Best, The Netherlands) at the Cardiovascular Center of our institution. The X-ray tubes of the device used for the procedure had focal sizes of 0.5 mm and 0.8 mm, with a maximum output of 125 kVp. Image acquisition was performed using auto exposure control at tube voltages of 75 kVp and 80 kVp in an image acquisition mode of 15 frames/s. To minimize interoperator variability, all collected examinations were performed by six cardiac interventional specialists certified by the Korean Society of Cardiac Interventional Procedures, who had over 8 years of clinical experience in cardiac interventional procedures. The scope of data collection included patients aged ≥20 years who weighed between 55.2 kg and 73.95 kg, corresponding to the 25th–75th percentile in the 8th Korean Standard Body Type Survey [16].

### 2.2. Dosimetric Data Extraction

Radiation dose data were retrospectively extracted by analyzing the DICOM Radiation Dose Structured Reports (RDSRs) stored in the picture archiving and communication system. The RDSRs were automatically generated by the angiographic system following each procedure and contained comprehensive dosimetric parameters, including the total KAP, exposure cumulative KAP, exposure series, exposure images, fluoroscopy cumulative KAP, and total air kerma.

### 2.3. Data Analysis and DRL Establishment

In this study, the total KAP was used as the primary dosimetric indicator. All KAP values were converted to Gy·cm^2^ for consistent analysis. In addition, the total fluoroscopy time was collected and evaluated as a surrogate metric to assess the procedural complexity.

The procedures were categorized according to the classification used by the Korean national DRLs, as shown in Table 1. Descriptive statistics, including the mean, median (50th percentile), 1st quartile (25th percentile), and 3rd quartile (75th percentile), were calculated for each procedural category. In accordance with the recommendations of the International commission on Radiological Protection, institutional DRLs were set at the 75th percentile of the total KAP distribution for each specific procedure.

## 3. Results

### 3.1. Establishment of Institutional DRLs

**Figure 1:**
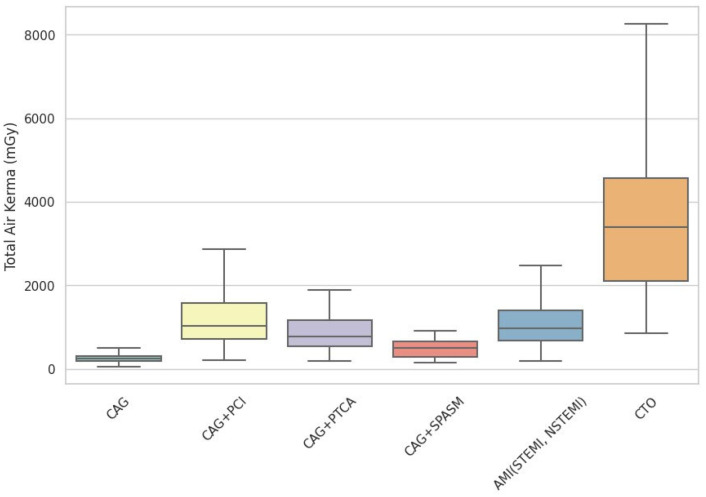
The distribution of total air kerma for each group (mGy). CAG, coronary angiography; PCI, percutaneous coronary intervention; PTCA, percutaneous transluminal coronary angioplasty; SPASM, coronary artery vasospasm; AMI, acute myocardial infarction; STEMI, ST-elevation myocardial infarction; NSTEMI, non-ST-elevation myocardial infarction; CTO, chronic total occlusion.

**Figure 2:**
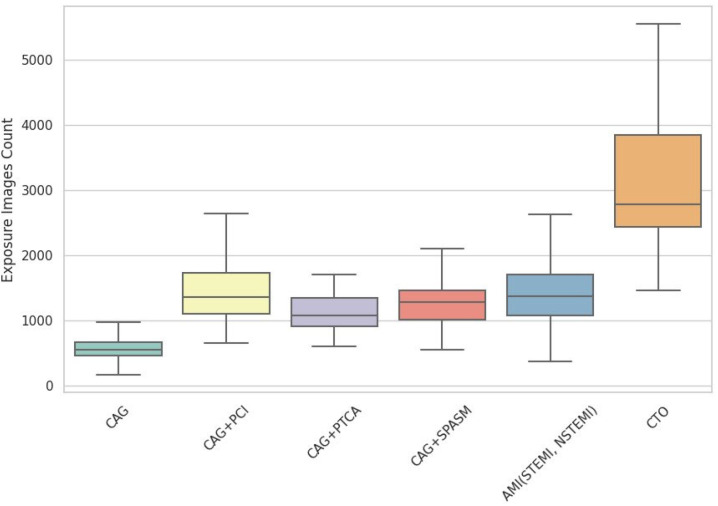
The number of image acquisitions for each group. CAG, coronary angiography; PCI, percutaneous coronary intervention; PTCA, percutaneous transluminal coronary angioplasty; SPASM, coronary artery vasospasm; AMI, acute myocardial infarction; STEMI, ST-elevation myocardial infarction; NSTEMI, non-ST-elevation myocardial infarction; CTO, chronic total occlusion.

On the basis of an analysis of 1663 RDSRs, institutional DRLs (75th percentile) were established for various cardiovascular procedures. Table 2 presents the DRLs for total KAP values for each procedure. The value was the lowest for diagnostic CAG (23.43 Gy·cm^2^) and the highest for chronic total occlusion (CTO) interventions (329.45 Gy·cm^2^). Intermediate values were observed for CAG + coronary artery vasospasm (SPASM) (50.60 Gy·cm^2^), CAG + percutaneous transluminal coronary angioplasty (PTCA) (87.58 Gy·cm^2^), CAG + acute myocardial infarction (AMI) (96.87 Gy·cm^2^), and CAG + percutaneous coronary intervention (PCI) (103.17 Gy·cm^2^). The markedly elevated dose in the CTO group confirmed that procedural complexity was a critical determinant of the radiation burden on the patient. Figure 1 shows the distribution of total air kerma for each group, while Figure 2 shows the number of image acquisitions for each group.

### 3.2. Analysis of Fluoroscopy Dose and Contribution

Table 3 presents the cumulative fluoroscopy KAP values, which ranged from 6.98 Gy·cm^2^ for CAG to 219.30 Gy·cm^2^ for CTO. An analysis of the dose components revealed distinct patterns; for diagnostic or simple procedures (CAG + CAG, CAG + SPASM), the radiation dose was predominantly driven by cine image acquisition. In contrast, for moderate-complexity interventions such as CAG + PCI, CAG + PTCA, and AMI, the contribution of fluoroscopy became more significant, showing a balanced proportion with cine image acquisition.

### 3.3. Fluoroscopy Time Characteristics

The distribution of fluoroscopy times (Table 4) paralleled the total KAP values. While CAG required minimal time (DRL, 3.8 min), thus limiting radiation exposure, therapeutic interventions required significantly longer durations. The DRLs for CAG + PCI, CAG + PTCA, and AMI ranged between approximately 18 and 25 min (25.0, 17.8, and 20.45 min, respectively). The CTO group, which required the most intricate manipulation, had a prolonged fluoroscopic time of 73.03 min.

### 3.4. Dose Comparison Between Single-Vessel and Multivessel Interventions

Among 657 CAG + PCI procedures, the 75th percentile of the total KAP was 83.49 Gy·cm^2^ for single-vessel procedures, 156.61 Gy·cm^2^ for multivessel procedures, and 103.17 Gy·cm^2^ for all procedures. The cumulative fluoroscopy KAP was 46.66 Gy·cm^2^ for single-vessel procedures, 101.06 Gy·cm^2^ for multivessel procedures, and 60.77 Gy·cm^2^ for all procedures. The fluoroscopy time was 18.68 min for single-vessel procedures, 37.9 min for multivessel procedures, and 25.0 min for all procedures.

### 3.5. Radiation Dose Characteristics in AMI Procedures

In a cohort of 449 AMIs (STEMI, NSTEMI), the 75th percentile of the total KAP was 88.80 Gy·cm^2^ for single-vessel procedures, 172.05 Gy·cm^2^ for multivessel procedures, and 96.8 Gy·cm^2^ for all procedures. The DRL for the fluoroscopy time was 17.5 min for single-vessel procedures, 38.0 min for multivessel procedures, and 20.45 min for all procedures.

### 3.6. Radiation Dose Characteristics in CTO Procedures

In an analysis of 58 CTO procedures, the 75th percentiles of the total KAP were 306.12, 470.26, and 329.45 Gy·cm^2^ for single-vessel, multivessel, and all procedures, respectively. The value for multivessel procedures was approximately 1.5 times higher than that for single-vessel procedures; this confirmed that an increase in the number of lesions contributed directly to higher patient radiation exposure. The DRLs for cumulative fluoroscopy KAP were 178.78, 303.03, and 219.29 Gy·cm^2^ for single-vessel, multivessel, and all procedures, respectively, with the multivessel group displaying a value approximately 1.7 times higher than that for the single-vessel group. Regarding the fluoroscopy time, the DRLs were established as 70.7, 101, and 73.03 min for single-vessel, multivessel, and all procedures, respectively. These findings indicated that CTO procedures typically required prolonged fluoroscopy exceeding 1 h on average. Specifically, multivessel CTO required prolonged fluoroscopy exceeding 100 min. A comparison between the total and cumulative fluoroscopy KAPs revealed that fluoroscopy accounted for approximately 58%, 64%, and 67% of the total radiation dose in the single-vessel, multi-vessel, and overall patient cohorts, respectively.

## 4. Discussion

In this study, we analyzed radiation exposure doses in 1663 cardiovascular angiography and interventional procedures at a single institution in order to establish institutional DRLs. When comparing these established DRLs with the 2025 national DRLs published by the Korea Disease Control and Prevention Agency, the total KAP for CAG was 25.4% higher, while the fluoroscopy time was 47.2% lower. This discrepancy indicates that high-resolution cine imaging for image recording accounts for a significant proportion of the total radiation dose in CAG. Although improved operator proficiency and standardized approaches have successfully reduced fluoroscopy times by minimizing unnecessary real-time imaging, the repeated high-definition cine runs required to secure diagnostic information deliver a much higher dose per unit time, acting as the primary driver of the overall radiation dose increase in CAG.

It is suggested that modern angiographic equipment settings such as high-resolution imaging modes and increased frame acquisition rates contribute to higher radiation doses during image recording. Consequently, this appears to have resulted in a paradoxical trend where the total KAP has increased despite a reduction in the fluoroscopy time.

Conversely, the DRLs for interventional procedures exceeded the national DRLs for all parameters. For PCI, the DRLs for the total KAP and fluoroscopy time increased by 62.7% and 24.4%, respectively, compared with domestic standards. A provocation test for SPASM also demonstrated a substantial increase, with the DRLs for the total KAP and fluoroscopy time exceeding the national guidelines by 98.9% and 53.4%, respectively. This indicated that significant radiation exposure can occur even during certain diagnostic tests. Similarly, for AMI procedures, the DRLs for the total KAP and fluoroscopy times were 65.5% and 32.2% higher, respectively. CTO interventions recorded the highest exposure levels, with the DRLs for the total KAP and fluoroscopy times exceeding the national DRLs by 208.4% and 56.8%, respectively; these figures were approximately 14 and 19 times higher than those for CAG, respectively, reflecting the clinical reality of CTO interventions, which inherently require a heavy dependence on fluoroscopy for approaching complex lesions.

Table 5 compares the 2025 Korean national DRLs for cardiovascular interventions with those in several other countries. When comparing our institutional DRLs with these international standards, contrasting trends emerged depending on procedural complexity. For CAG, the DRL for the total KAP was 24.4%, 33%, and 50.1% lower than those in the UK, Europe, and Japan, with fluoroscopy times also being generally well managed. In contrast, the DRL for the total KAP in PCI was 118.9% higher than that in the UK and 21.3% higher than that in Europe, although it remained within a range similar to Japan’s DRL. Furthermore, for CTO interventions, the DRL for the total KAP in this study was 64.7% higher than that in Japan; this confirmed that highly complex interventional procedures result in substantial radiation exposure.

Onn the basis of our findings, the respective contributions of fluoroscopy and cine imaging to the total radiation dose distinctly varied according to the difficulty of the procedure. In CAG and SPASM, cine imaging had a greater contribution, ranging from 55% to 60% of the total dose. In contrast, for complex procedures such as PCI, PTCA, AMI, and CTO interventions, real-time fluoroscopy accounted for over 50% of the total KAP, peaking at 67% for CTO interventions. This shift is attributable to an increased reliance on real-time fluoroscopic guidance necessitated by the complexity of the lesion approach and procedure progression, underscoring the critical need for proactive implementation of fluoroscopy-specific dose reduction strategies during highly complex procedures. Ultimately, these results demonstrate that radiation exposure increases exponentially, rather than linearly, as procedural complexity increases.

Furthermore, a comparative analysis between single- and multivessel procedures revealed that multivessel interventions exhibited values more than twice as high as those exhibited by single-vessel interventions in terms of the total KAP, cumulative fluoroscopy KAP, and fluoroscopy time. Notably, the proportion of fluoroscopy procedures within the total radiation dose increased from approximately 50% in the single-vessel group to >65% in the multivessel group. These results quantitatively confirm that fluoroscopy dependence increases with increasing lesion numbers, subsequently increasing the required radiation dose for a patient. This study reinforces that procedural complexity represents a decisive factor in patient exposure during cardiovascular interventions, highlighting the necessity for dose management tailored to specific procedure types. Specifically, for simple CAG, minimizing the frequency of high-resolution image acquisitions is the primary step for dose reduction. In contrast, for difficult procedures such as multivessel PCI, AMI, and CTO, multifaceted efforts including shortening of the fluoroscopy time, use of low-dose modes, a reduction in frame rates, and application of an appropriate field of view are required.

The institutional DRLs presented in this study exhibited an excellent level of optimization for simple diagnostic procedures. However, for complex interventional procedures, radiation doses were observed to exceed both domestic and international DRLs. These findings reflect institutional clinical characteristics such as patient severity, procedural difficulty, and variations in equipment protocols. While medical institutions should use national DRLs as benchmarks, they must establish and update their own institutional DRLs based on local data. For high-difficulty cases like CTO in particular, radiation reduction strategies such as preprocedural planning, use of low-dose fluoroscopy modes, minimization of unnecessary imaging, and enhancement of operator education and feedback must be actively and systematically implemented.

Cardiovascular interventions inherently involve significant radiation exposure because of their reliance on high-resolution imaging. The amount of radiation dose could vary substantially depending on several factors, including the experience of the operator, imaging equipment performance, and specific characteristics of the patient lesions.

Consequently, establishment of institution-specific DRLs and their comparison with both domestic and international standards provides essential baseline data for the optimization of radiation protection and dose management.

National DRLs should serve as benchmarks for comparison with local or institutional data and must be periodically updated. Local DRLs reflecting the unique characteristics and environment of each medical facility serve as definitive starting points for achieving concrete and realistic radiation dose optimization in clinical practice.

According to the findings of this study, reducing the patient radiation dose in clinical practice requires not only shortening of the fluoroscopy time but also active utilization of device settings such as lower frame rate configurations, minimal high-resolution image acquisition, and storage of low-resolution fluoroscopic images. Patient radiation exposure can be reduced by half simply by reducing the image acquisition setting from 15 frames/s to 7.5 frames/s. Increasing the distance between the X-ray tube and the patient, minimizing the distance between the X-ray detector and the patient, and minimizing image magnification can also actively reduce patient radiation dose. Furthermore, it is essential to establish quality control procedures that precisely reflect procedural difficulty and lesion complexity in institutional DRL settings. These should be periodically evaluated against national or regional standards. Ultimately, continuous efforts to minimize radiation exposure must be sustained through close collaborations among medical staff.

### Limitation

This study compared the national DRL based on the case of a single medical institution.

## 5. Conclusions

The results of this study confirmed that simple procedures such as CAG and SPASM require radiation levels similar to or lower than domestic and internationally reported values, indicating relatively optimized radiation management. Conversely, complex interventional procedures, including PCI, PTCA, AMI, and CTO, yielded values exceeding both domestic and international DRLs, with CTO procedures recording the highest patient radiation exposure. A similar trend was observed in fluoroscopy time analysis. While the fluoroscopy time for CAG was relatively short compared with international standards, those for PCI and AMI were similar to or slightly longer than domestic and international benchmarks. However, the fluoroscopy time for CTO significantly exceeded the domestic benchmark. Although constrained by the limited number of cases from a single medical institution, the findings of this study clearly demonstrate that procedural difficulty and complexity directly increase radiation exposure doses. By establishing single-institution DRLs and comparing them with domestic and international standards, this study provides essential baseline data for medical institutions to effectively manage patient radiation exposure. Ultimately, these findings can be used for future safety management initiatives through continuous comparisons with DRLs from various countries, and they are expected to contribute significantly to the development of radiation optimization guidelines aimed at enhancing patient safety.

## Figures and Tables

**Table 1:** Categorization of procedures according to the classification used by the Korean national Diagnostic Reference Levels.

Abbreviation	Procedure Name
CAG	Coronary Angiography
PCI	Percutaneous Coronary Intervention
PTCA	Percutaneous Transluminal Coronary Angioplasty
SPASM	Coronary Artery Vasospasm
AMI	Acute Myocardial Infarction
STEMI	ST-Elevation Myocardial Infarction
NSTEMI	Non-ST Elevation Myocardial Infarction
CTO	Chronic Total Occlusion

**Table 2:** Diagnostic reference levels for total KAP values (Gy·cm^2^) for each procedure.

	Mean	Minimum	25th %ile	50th %ile	75th %ile	Maximum
CAG	19.44	3.65	12.98	17.19	23.43	82.38
CAG + PCI	84.79	15.72	46.53	68.33	103.17	388.34
CAG + PTCA	71.18	13.82	34.47	56.58	87.58	255.66
CAG + SPASM	36.4	8.66	21.27	37.03	50.6	65.22
AMI (STEMI, NSTEMI)	81.25	11.52	46.62	67.6	96.87	691.11
CTO	257	79.62	143.9	208.78	329.45	822.87

KAP, kerma-air product; CAG, coronary angiography; PCI, percutaneous coronary intervention; PTCA, percutaneous transluminal coronary angioplasty; SPASM, coronary artery vasospasm; AMI, acute myocardial infarction; STEMI, ST-elevation myocardial infarction; NSTEMI, non-ST-elevation myocardial infarction; CTO, chronic total occlusion.

**Table 3:** Diagnostic reference levels for cumulative fluoroscopy KAP (Gy·cm^2^) values for each procedure.

	Mean	Minimum	25th %ile	50th %ile	75th %ile	Maximum
CAG	6.15	1.05	2.93	4.34	6.98	48.17
CAG + PCI	49.82	4.81	22.28	36.57	60.77	301.24
CAG + PTCA	40.6	6.12	15.26	27.14	45.83	153.07
CAG + SPASM	10.84	2.6	6.54	9.53	13.2	28.73
AMI (STEMI, NSTEMI)	44.9	4.57	22.75	33.1	51.81	447.27
CTO	171.3	39.62	86.7	136.74	219.3	673.78

KAP, kerma-air product; CAG, coronary angiography; PCI, percutaneous coronary intervention; PTCA, percutaneous transluminal coronary angioplasty; SPASM, coronary artery vasospasm; AMI, acute myocardial infarction; STEMI, ST-elevation myocardial infarction; NSTEMI, non-ST-elevation myocardial infarction; CTO, chronic total occlusion.

**Table 4:** Diagnostic reference levels for the fluoroscopy time (min) in each procedure.

	Mean	Minimum	25th %ile	50th %ile	75th %ile	Maximum
CAG	3.13	1	1.6	2.45	3.8	22.7
CAG + PCI	20.38	3.4	11.1	16	25	136
CAG + PTCA	16.01	3.2	8.1	12.1	17.8	72.6
CAG + SPASM	5.57	2.1	3.68	4.5	8.3	10.6
AMI (STEMI, NSTEMI)	17.4	4.3	10.5	13.9	20.45	131
CTO	57.46	13.3	32.1	51.65	73.03	177

CAG, coronary angiography; PCI, percutaneous coronary intervention; PTCA, percutaneous transluminal coronary angioplasty; SPASM, coronary artery vasospasm; AMI, acute myocardial infarction; STEMI, ST-elevation myocardial infarction; NSTEMI, non-ST-elevation myocardial infarction; CTO, chronic total occlusion.

**Table 5:** Comparison between Korean national DRLs and international DRLs for coronary angiography and interventional cardiology procedures.

Examination	Korea (2025) [14]		UK (2010, HPA-CRCE-034) [17]		Spanish (2020) [18]		Europe (2018) [19]		Finland (2019) [20]		Japan (2025) [21]	
	KAP	FlT	KAP	FlT	KAP	FlT	KAP	FlT	KAP	FlT	KAP	FlT
CAG	18.68	7.2	31	4.3	39	6.7	35		30	6	47	
CAG + PCI	63.4	20.01							75	18.4	100	
CAG + PTCA	53.89	21.24	40 *	11.3 *	78	15						
CAG + SPASM	25.44	5.41										
AMI (STEMI, NSTEMI)	58.52	15.47										
CTO	106.83	46.59					137				200	
PCI	49.94	24.39					85					
TAVA							130		90	21.5	78	

* Single-stent PTCA. DRL, Diagnostic Reference Level; CAG, coronary angiography; PCI, percutaneous coronary intervention; PTCA, percutaneous transluminal coronary angioplasty; SPASM, coronary artery vasospasm; AMI, acute myocardial infarction; STEMI, ST-elevation myocardial infarction; NSTEMI, non-ST-elevation myocardial infarction; CTO, chronic total occlusion; KAP, kerma-area product; FLT, fluorometry.

## Data Availability

The data presented in this study are available upon request from the corresponding author due to privacy and ethical restrictions.

## Abbreviations

The following abbreviations are used in this manuscript:

DRLs	Diagnostic Reference Levels
RDSR	Dose Structured Reports
KAP	Kerma-area product
CAG	Coronary Angiography
PCI	Percutaneous Coronary Intervention
PTCA	Percutaneous Transluminal Coronary Angioplasty
SPASM	Coronary Artery Vasospasm
AMI	Acute Myocardial Infarction
STEMI	ST-Elevation Myocardial Infarction
NSTEMI	Non-ST Elevation Myocardial Infarction
CTO	Chronic Total Occlusion
